# Effect of Blood Vessel Segmentation on the Outcome of Electroporation-Based Treatments of Liver Tumors

**DOI:** 10.1371/journal.pone.0125591

**Published:** 2015-05-05

**Authors:** Marija Marčan, Bor Kos, Damijan Miklavčič

**Affiliations:** University of Ljubljana, Faculty of Electrical Engineering, Ljubljana, Slovenia; University of Nebraska Medical Center, UNITED STATES

## Abstract

Electroporation-based treatments rely on increasing the permeability of the cell membrane by high voltage electric pulses applied to tissue via electrodes. To ensure that the whole tumor is covered with sufficiently high electric field, accurate numerical models are built based on individual patient anatomy. Extraction of patient's anatomy through segmentation of medical images inevitably produces some errors. In order to ensure the robustness of treatment planning, it is necessary to evaluate the potential effect of such errors on the electric field distribution. In this work we focus on determining the effect of errors in automatic segmentation of hepatic vessels on the electric field distribution in electroporation-based treatments in the liver. First, a numerical analysis was performed on a simple 'sphere and cylinder' model for tumors and vessels of different sizes and relative positions. Second, an analysis of two models extracted from medical images of real patients in which we introduced variations of an error of the automatic vessel segmentation method was performed. The results obtained from a simple model indicate that ignoring the vessels when calculating the electric field distribution can cause insufficient coverage of the tumor with electric fields. Results of this study indicate that this effect happens for small (10 mm) and medium-sized (30 mm) tumors, especially in the absence of a central electrode inserted in the tumor. The results obtained from the real-case models also show higher negative impact of automatic vessel segmentation errors on the electric field distribution when the central electrode is absent. However, the average error of the automatic vessel segmentation did not have an impact on the electric field distribution if the central electrode was present. This suggests the algorithm is robust enough to be used in creating a model for treatment parameter optimization, but with a central electrode.

## Introduction

Exposing a biological cell to a sufficiently high electric field causes increased permeability of the cell membrane. This increased permeability of the membrane allows transfer of molecules which normally lack membrane transport mechanisms into the cell. The described effect of the electric field on the cell is called electroporation [1, 2]. Electroporation can be either reversible or irreversible. The reversible/irreversible nature of electroporation is strongly dependent on pulse amplitude, duration, and number of pulses [3]. In reversible electroporation, the cell membrane eventually returns to its normal state. Irreversible electroporation however leads to cell death because the cell membrane is permanently disrupted or due to the extensive loss of the intracellular components [4]. Combination of reversible electroporation with traditional methods of chemotherapy has resulted in a technology for tumor treatment named electrochemotherapy (ECT) [5, 6]. Irreversible electroporation (IRE) has found its application in tumor treatment as a tissue ablation procedure, its main advantage being the fact that it does not rely on thermal effects to achieve tissue ablation [7, 8].

In order for the tumor treatments based on electroporation to be successful the whole tumor must be covered by a sufficiently high electric field [9]. The magnitude and distribution of the electric field depends on the number and the position of the electrodes, the amplitudes of pulses applied per electrode pair and the electric properties of the tissue, especially conductivity [10, 11]. Ensuring the complete tumor coverage with a sufficiently high electric field is challenging in the case of deep-seated solid tumors as well as large tumors [12–14]. Predictability of an adequate distribution of the electric field can be best achieved by calculating a patient-specific treatment plan as a part of an electroporation-based treatment procedure [15–17].

A patient-specific treatment plan for electroporation-based treatment of deep-seated solid tumors takes into account patient geometry and tissue properties to generate an optimal set of treatment parameters [18, 19]. The patient model is built by segmenting the medical images and then used to perform numerical calculations of the electric field distribution. First use of the treatment planning procedure was done in a patient with a metastasis in the thigh [15], then the procedure was upgraded in a clinical study of liver metastases [20]. For the purpose of treating the colorectal metastases by ECT, an algorithm for automatic segmentation of the liver from MRI images was developed [21], as well as an algorithm for segmentation of hepatic vessels reported in our previous work [22].

Segmentation of medical images is susceptible to errors, which must be taken into account when analyzing robustness of the treatment plan. In order to evaluate the effect of errors in segmentation of hepatic vessels we performed studies consisting of numerical modeling of the electric field distribution in ECT and IRE of a simplified model of tumor and vessel, and ECT of two models obtained from two real patients. The first part of the studies focused on determining in what measure the vessels of different sizes influence the distribution of the electric field in an already optimized model. Through these experiments we aimed to establish if ignoring the vessels in the numerical calculations could potentially have negative effects on the tumor coverage with a sufficiently high electric field. The second part of the studies observed the effect of known errors of the vessel segmentation method reported in our previous work [22] on possible over- or underestimation of the electric field distribution which leads to over- or under-treatment.

## Materials and Methods

### Simplified model

**Fig 1:**
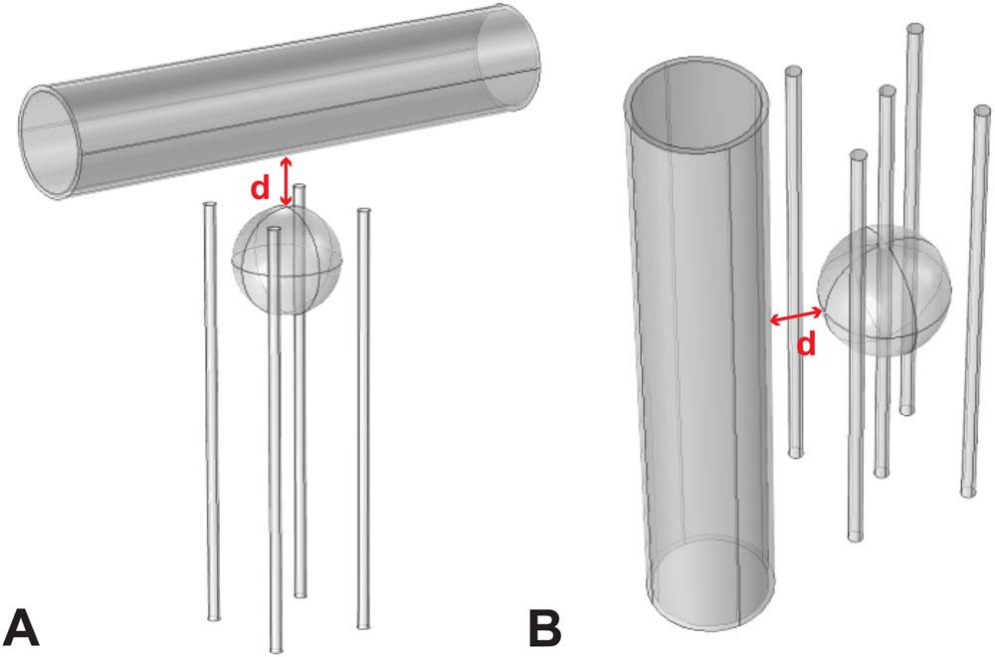
Two different electrode configurations and two different positions of the vessel. A: 4 electrodes with vessel perpendicular to them. B: 5 electrodes with vessel parallel to them. “d” denotes distance from vessel to tumor.

The simplified model consisted of a sphere and a cylinder, where the sphere represented the tumor and the cylinder represented the vessel. We used three different sphere diameters: 10 mm, 30 mm and 50 mm. Such choice of sphere size was made because colorectal metastases in the liver that are most often treated with ECT have a diameter 10–30 mm [20], while primary tumors in the liver can be as large as 50 mm. The vessel that was included in the model varied in size and position relative to the tumor. The diameters of the vessel were 1 mm, 3 mm, 5 mm, 10 mm and 15 mm, which are all actual sizes of hepatic vessels. The vessel was positioned in two different orientations, as shown in Fig 1: perpendicular (A) and parallel to the electrodes (B). Additionally, we varied the distance of the vessel from the tumor, setting it to 0 mm, 1 mm, 3 mm, 5 mm and 10 mm. These variations in vessel size and position were applied on all combinations of tumor size and treatment type as indicated in Table 1. The vessel was modeled as a combination of blood tissue and vessel wall tissue which had a thickness of 10% of the vessel diameter. Such vessel structure is observed in veins, which make up the majority of large hepatic vessels (vena porta, vena cava and main hepatic veins) [23].

**Table 1:** Electrode number and position per tumor size and treatment type.

**Tumor diameter size [mm]**	**Treatment**	**Electrode number**	**Electrode position**
10	ECT	4	4 in a box around the tumor, 2 mm away from the tumor edge
10	ECT	5	4 in a box around the tumor, 2 mm away from the tumor edge + 1 in the center
30	ECT	5	4 in a box around the tumor, 2 mm away from the tumor edge + 1 in the center
50	ECT	7	6 in a hexagon around the tumor, 2 mm away from the tumor edge + 1 in the center
10	IRE	4	4 in a box around the tumor, 2 mm away from the tumor edge
10	IRE	5	4 in a box around the tumor, 2 mm away from the tumor edge + 1 in the center
30	IRE	7	6 in a hexagon inside the tumor, 7 mm away from the tumor center + 1 in the center

For all three tumor sizes we used electrodes of 1.2 mm diameter with an active tip of 40 mm length. The number and the position of the electrodes varied depending on the tumor size and the type of the electroporation-based treatment (ECT or IRE). The information about number and the position of the electrodes per combination of tumor size and treatment is presented in Table 1. Generally, the depth of the electrode relative to tumor was such that the tumor was positioned in the middle of the active part of the electrodes. The only exceptions to this rule were made in cases of a 10 mm tumor and large vessels (10 mm and 15 mm diameter) perpendicular to the electrodes, where the depth was adjusted in order to avoid electrodes penetrating the vessels. In such cases, the depth of the electrodes was set to 1 or 5 mm further than the edge of the tumor that is near the vessel. Complete geometry was transformed into a mesh of free tetrahedral elements using the user-controlled meshing sequence of COMSOL Multiphysics (COMSOL, Stockholm, Sweden) with element size set to ‘Fine’ according to COMSOL properties and values of all other properties left default. The number of elements ranged from 50000 to 70000, based on the specific geometry.

All models included a dynamic change in the conductivity of all tissues that occurs due to electroporation [19, 24, 25]. The conductivity change was modeled as a smoothed Heaviside function of the electric field in the range between the threshold of reversible electroporation, E_0_ and the irreversible threshold, E_1_ [24, 25]. Values of the above mentioned thresholds for liver and tumor tissue were taken from literature [26], while for the vessel wall and the blood they were estimated based on the experiments performed on similar tissue [27, 28]. We have also used different initial and final conductivities, *σ*_0_ and *σ*_1_ for each of the tissues [26–31]. The values of E_0_ and E_1_ along with initial and final conductivities *σ*_0_ and *σ*_1_ for each tissue are given in Table 2. It should be noted that while the conductivity increase for liver and tumor tissues was already determined by measurements [28, 29], for vessel wall and blood tissue we had to extrapolate the data from measurements on similar tissue. Therefore, for vessel walls we have chosen the conductivity increase factor of 3 based on experiments made with muscle tissue [28]. For blood we first made the assumption that it is in fact a suspension of red blood cells in a high conductive medium (plasma), with a normal cell volume fraction of 0.46. We then referred to the previous work of Pavlin et al. on cell suspension electroporation [27, 32] and determined that factor of conductivity for blood with given assumptions is around 0.5.

**Table 2:** Values of electroporation thresholds and conductivities for different tissues with corresponding sources.

**Tissue**	**E_0_ [V/cm]**	**E_1_ [V/cm]**	**σ_0_ [S/m]**	**σ_1_ [S/m]**
Liver	350 [28]	700 [28]	0.04 [29]	0.12 [26, 28, 29]
Tumor	400 [26]	800 [26]	0.2 [30]	0.7 [26, 28, 30]
Vessel wall	400 [28]	800 [28]	0.26 [31]	0.78 [25, 26, 28, 31]
Blood	400 [27]	1100 [27]	0.7 [31]	1.05 [27, 31, 32]

For all the models we calculated the electric field distribution using finite element analysis in COMSOL Multiphysics (COMSOL, Stockholm, Sweden). From the resulting electric field distribution we extracted the percentage of tumor volume covered with the electric field higher than the target field, which is 400 V/cm for ECT and 600 V/cm for IRE. The values of target fields are determined based on the definition that a successful electroporation-based treatment achieves above 99.9% cell kill. The statistical model of cell kill due to electroporation was previously described in the work of Golberg et al. [33] and states that cell kill depends not only on the electric field but also on number of pulses and pulse duration. Depending on number of pulses used the target field that is necessary to achieve 99.9% cell kill can thus be lower than E_1_. In our case the target field values are set with respect to the number of pulses typically used for different electroporation-based treatment, which is 8 for ECT and 90 for IRE.

The workflow of the numerical calculations was divided in two parts. Firstly, we observed only the model of the tumor without the vessel, on which we employed the optimization algorithm described in the work of Zupanic et al [19]. The optimization was performed with respect to the voltages between neighboring (outer) pairs of electrodes and between the electrodes in diagonal direction. The geometry of the model, along with the electrode positions, was kept constant. The optimization criterion was to achieve 100% coverage of the tumor with a sufficiently high electric field (400 V/cm for ECT or 600 V/cm for IRE), while minimizing the amount of liver tissue covered by the electric field above the IRE threshold for liver (400 V/cm) [33, 34]. Secondly, we included the vessel into the model and performed the calculation of the electric field distribution with the same values of voltages that were optimal for the model without the vessel. We then determined the percent of the tumor that was covered with a sufficiently high electric field. In this way, the difference between a model that does not take vessels into account and the one that does was obtained. The above described workflow was performed for all combinations of tumor size and treatment type indicated in Table 1. and for all variations in vessel size and position.

### Real patient model

Two models used in the second part of the studies were obtained from patients that were treated with ECT for a colorectal liver metastasis. The first patient was also reported in the work of Edhemovic et al. [16, 20]. Five years after the ECT that patient is alive and disease-free.

**Fig 2:**
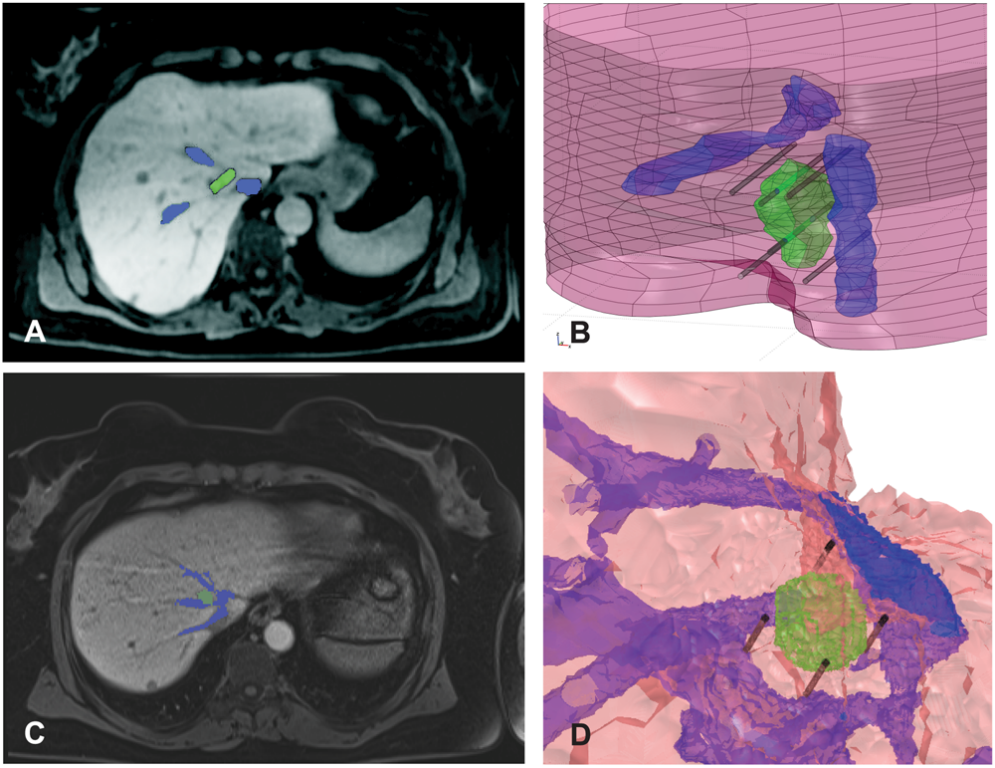
Real patient cases used in our study. A: MRI of the liver for first patient case. B: Reconstructed 3D model of the first case with inserted electrodes. C: MRI of the liver for second patient case. D: Reconstructed 3D model of the second case with inserted electrodes. In all images the structure colored green is the tumor, while the nearby major vessels are colored blue.

Specificity of these particular cases is that the tumor was located in the close vicinity of major hepatic vessels: vena cava and the second branches of the hepatic vein. One MRI slice along with the reconstructed 3D model for each case are shown in Fig 2. The sizes of the tumors, as determined by the radiologist, were 35 mm × 20 mm for the first tumor and 15 mm × 11 mm for the second tumor.

The 3D models of the tumors and the nearby vessels were obtained based on the segmentation of the MRI images by an expert radiologist. The number of the electrodes used in the two cases varied due to different tumor sizes. For the first case, the actual treatment was performed using six individual electrodes: two inside the tumor and four outside. We kept the same electrode configuration in our model. For the second case, the actual treatment was performed using five individual electrodes: one inside the tumor and four outside. In our model we used the same five electrode configuration and additionally created a model with only four outer electrodes (without the central electrode in the tumor). Therefore, we had three models in total: one model for the first case and two for the second case.

In all three models we varied the size and the position of the vessels in order to include the error of the automatic vessel segmentation algorithm. The mentioned algorithm for automatic segmentation of hepatic vessels from MRI images was described and validated in our previous work [22]. The results of the validation report an average error of the algorithm to be 0.9 pixel, while the maximum error is 2.8 pixels. These values of the error were introduced in the model through six transformations: enlarging, shrinking, shift left, shift right, shift up and shift down. The values used in the transformations were rounded from 0.9 and 2.8 to 1 and 3, respectively, in order to use integers.

The electrodes were initially positioned at the coordinates used in the actual treatments. However, in the models with modified vessel size and position it could happen that the initial electrode trajectory would go through the vessel, which should be avoided when possible, since it can cause failure of pulse delivery due to excessive currents. Namely, the currently available ECT and IRE pulse generator have a 50 A maximum current limit. The high conductivity of blood can cause currents to rise above that limit, which causes the generator to abort pulse delivery to that particular electrode pair. We have therefore visually inspected images of all models with modified vessels and minimally adjusted the electrode positions to avoid having an electrode go through the vessel.

The workflow of the numerical calculations in this part of the study was reversed in respect to the workflow of the simplified model. Here we first introduced the error in vessel geometry, after which we ran the optimization of the treatment, resulting in optimal values of voltages per electrode pair. Again, only the voltages were optimized, while the positions of the electrodes were kept constant, as they were set in the original treatment of the patient. The obtained values of optimal voltages were then used in numerical calculations on an original model with correct vessel geometry. Based on the results of these calculations we then calculated the coverage of the tumor with a sufficiently high electric field and observed if there was any insufficient coverage of the tumor that could have happened due to errors in vessel segmentation. On the real patient geometries we performed only the calculations for ECT treatment.

Additionally, we also calculated the differences in the coverage of tumors from real patient models between correct vessel geometry and not taking the vessels into account at all. The workflow for these calculations was the same as described above, with the difference that in the first step instead of introducing an error in vessel geometry the vessels were simply excluded from the model.

### Ethics statement

The two patients whose medical images were used in the experiments were a part of a clinical study “Treatment of Liver Metastases with Electrochemotherapy (ECTJ)” (EudraCt no. 2008-008290- 54, registered at Clinicaltrials.gov no. NCT01264952). The study was prospective, phase I/II, conducted at the Institute of Oncology Ljubljana, Ljubljana, Slovenia. Regulatory approvals from the Institutional board, as well as from the Medical Ethics Committee of the Republic of Slovenia were obtained, under following document numbers: KME 45/09/08, 108/10/12, and 46/12/13. Written consents of the patients were obtained. The patients included in the clinical study were treated according to the principles expressed in the Declaration of Helsinki.

## Results

### Simplified model

The first step of the studies performed on the simplified model, optimization of the model without the vessels, gave optimal values of voltages. The positions of the electrodes were not optimized. The obtained voltages are presented in Table 3 for each individual combination of tumor size, treatment type and electrode number.

**Table 3:** Optimal voltages for a model without the vessel, per tumor size, treatment type and electrode number.

**Tumor diameter size [mm]**	**Treatment**	**Electrode number**	**Voltage between outer electrodes [V]**	**Diagonal voltage [V]**
10	ECT	4	600	1000
10	ECT	5	800	500
30	ECT	5	2300	1500
50	ECT	7	1000	3000
10	IRE	4	3000	900
10	IRE	5	1100	2500
30	IRE	7	2900	2700

Diagonal voltage stands for voltage between the center electrode and surrounding electrodes. In the case of a configuration with 4 electrodes diagonal voltage is the voltage between non-neighbor electrodes.

**Fig 3:**
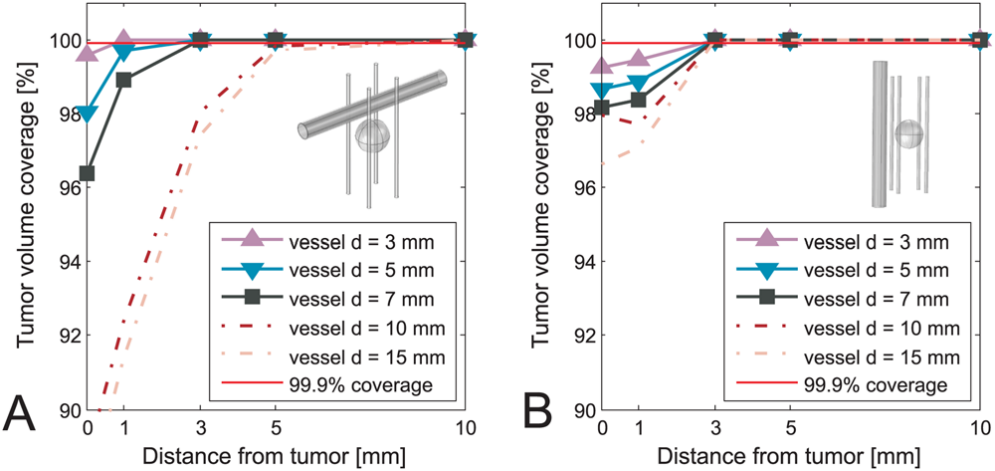
Tumor coverage for ECT of simplified model of 10 mm tumor with 4 electrodes. The coverage is plotted against different distances between vessel and tumor, and with respect to different vessel positions and sizes. A: Vessel perpendicular to the electrodes. B: Vessel parallel to the electrodes.

**Fig 4:**
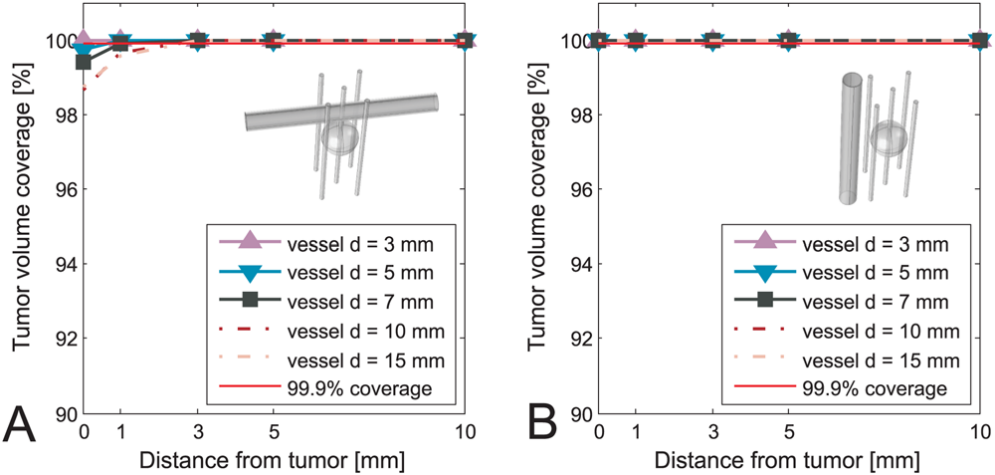
Tumor coverage for ECT of simplified model of 10 mm tumor with 5 electrodes. The coverage is plotted against different distances between vessel and tumor, and with respect to different vessel positions and sizes. A: Vessel perpendicular to the electrodes. B: Vessel parallel to the electrodes.

**Fig 5:**
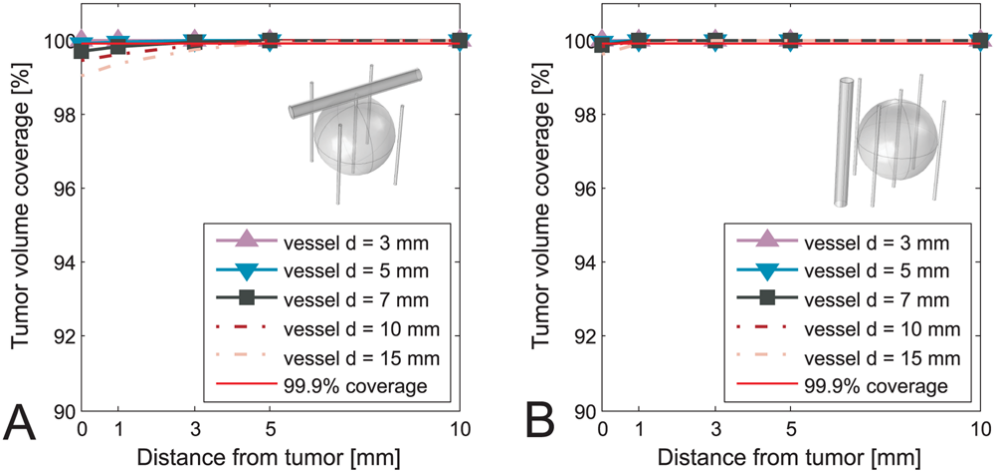
Tumor coverage for ECT of simplified model of 30 mm tumor. The coverage is plotted against different distances between vessel and tumor, and with respect to different vessel positions and sizes. A: Vessel perpendicular to the electrodes. B: Vessel parallel to the electrodes.

**Fig 6:**
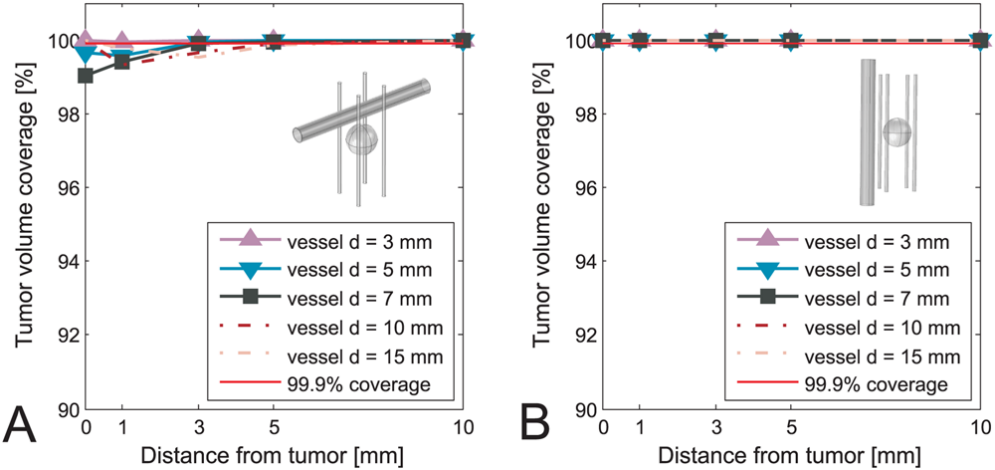
Tumor coverage for IRE of simplified model of 10 mm tumor with 4 electrodes. The coverage is plotted against different distances between vessel and tumor, and with respect to different vessel positions and sizes. A: Vessel perpendicular to the electrodes. B: Vessel parallel to the electrodes.

The values of voltages obtained from optimization as well as position of the electrodes were kept constant after including the vessels in the model. In Figs 3–6 we present results of the studies performed on a simplified model with both tumor and vessel. In all of the figures the coverage of the tumor volume by a sufficiently high electric field is plotted against the distance of the vessel from the tumor, grouped by different sizes of the vessel. The red line in all of the figures indicates a threshold of the tumor coverage required for a successful treatment, which is set to 99.9%. The choice of threshold being 99.9% was made for better readability of the figures and is otherwise in no way different than if we had chosen to set it to 100%. Each of the Figs 3–6 presents a different combination of tumor size, treatment type and electrode number. The figures are divided into an A. and B. part, where A. shows a case with vessel perpendicular to the electrodes while B. shows the case with vessel parallel to the electrodes. In cases of ECT of 50 mm tumor, IRE of 10 mm tumor with 5 electrodes and IRE of 30 mm tumor, the vessels have no negative effect on the tumor volume coverage at all. Therefore results of these cases are not graphically shown in this manuscript.

Based on results presented in Figs 3–6 neglecting vessels in model of the electric field distribution causes the tumor not to be entirely covered by sufficiently high electric field. The percentage of the tumor that is not covered with sufficiently high electric field leads to incomplete treatment, i.e. treatment failure. The extent of the negative effect of neglecting vessels depends on tumor size, vessel diameter, distance to tumor and orientation regarding the electrodes. For tumors with 10 mm diameter, the negative effect is observed for vessels with diameter 3–10 mm that are less than 3 mm away from the tumor, and vessels 10 mm or larger that are less than 5 mm away from the tumor. This observation was made for the worst case, which is ECT with 4 electrodes (Fig 3). For tumors with 30 mm diameter the worst case of the negative effect is again ECT, where the critical vessels have a diameter 7 mm and more and are within 5 mm distance to the tumor. In general, the negative influence of the vessels is directly proportional to vessel size and inversely proportional to vessel distance from the tumor. The only anomaly to this rule is observed for ECT and IRE of a 10 mm tumor performed with 4 electrodes, where the electrode depths (and subsequently optimal voltages) had to be changed for 10 mm and 15 mm vessels in order to avoid having all 4 electrodes go through the vessel, as described in the Materials and methods section. In the case of IRE of 10 mm tumor the breach of the vessel was inevitable up to 3 mm away from the tumor, which resulted in higher tumor coverage for 10 mm and 15 mm vessels than logically anticipated.

**Fig 7:**
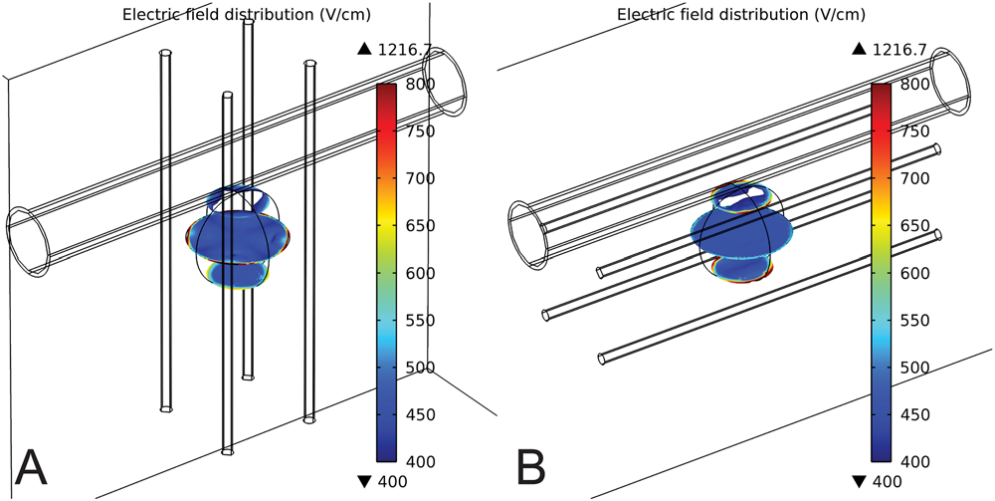
2D sliced color map of the electric field distribution for a case where a significant difference in tumor coverage is observed as a result of the presence of the blood vessel. The color map is presented for a simplified model of ECT of a 10 mm tumor with a 4 electrode configuration and in the presence of a 7 mm vessel which is 0 mm away from the tumor. The color maps are shown for two vessel positions: A: Vessel perpendicular to the electrodes. B: Vessel parallel to the electrodes. The slices for which the color map is shown span the plane parallel to the vessel. The relative position of the planes along the third axis (the one which the plane does not span) is the same in both cases and fixed at -4 mm, 0 mm, and 4 mm away from the tumor center.

The negative effect is observed in both ECT and IRE, although in IRE to a smaller extent, as is seen by comparing Figs 3 and 6. It is also significantly more pronounced in cases where all the electrodes are outside the tumor, and not so much if central electrode was present (Figs 3 vs. 4). Another interesting point to note by comparing the A and the B sides of all the Figs 3–6 is that the negative effect of the vessel on tumor coverage is higher when a vessel is perpendicular to the electrodes than when it is parallel to them. We also included a 2D color map of several slices for one of the simplified models in order to provide the visual insight at exactly how is the electric field distribution in tumor deformed due to presence of a vessel. The model in question is the one where the significant difference in tumor coverage was observed, namely it is the model of ECT of the 10 mm tumor with 4 electrode configuration and with a 7 mm vessel positioned at 0 mm away from the tumor. Fig 7 shows both the case where vessel is perpendicular to the electrodes (Fig 7A), and the case where vessel is parallel to the electrodes (Fig 7B). For better comparison of the impact of the vessel position with respect to the electrodes the planes in both Fig 7A and 7B are oriented parallel to the vessel. The relative position of the planes along the third axis (the one which the plane does not span) is the same in both cases and fixed at -4 mm, 0 mm, and 4 mm away from the tumor center.

### Real patient model

Results for the model of the first patient (larger tumor with six electrodes) did not show any negative effect on the coverage of the tumor volume for all possible types of segmentation errors that were observed in this study.

**Fig 8:**
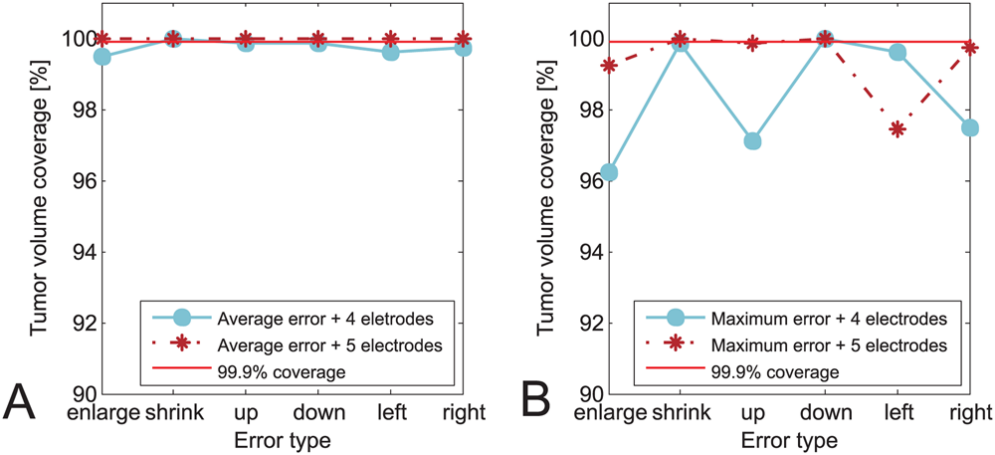
Tumor coverage for ECT of real case second patient with 15 mm × 11 mm tumor. The coverage is plotted against different types of transformations used to induce an error in vessel geometry and with respect to different sizes of the error. A: Configuration with four electrodes. B: Configuration with five electrodes.

Results for the two models of the second patient are shown in Fig 8. The figure shows the coverage of the tumor by a sufficiently high electric field against different types of transformations used to induce an error in vessel model that was used for optimization of treatment parameters. Fig 8A shows the results of the model where an average segmentation error (1 pixel) was introduced. Fig 8B shows the results of the model where the maximum segmentation error (3 pixels) was introduced. In both Fig 8A and 8B the results are grouped per two models: the model with four electrodes (no central electrode) and the model with five electrodes (with central electrode). It can be observed that the negative effect of the vessels is higher for maximum segmentation error than for average segmentation error, as would be expected. It is interesting to note that the negative effect of the maximum error is significantly larger in the case where no central electrode is present. Similar difference in the electrode configuration is also noted for average segmentation error. The difference for the average error is that the negative effect is minimal for the case of no central electrode and completely non-existent when the central electrode is present.

The results of comparing the real patient models with correct vessel geometry to real patient models where vessels were not taken into account differed between the two observed patients. For the first patient there was no difference in tumor coverage with a sufficiently high electric field if the vessels were not taken into account during the numerical optimizations of the voltages. For the second patient, if the vessels are not taken into account during optimizations the resulting coverage of the tumor would be 97.22% for the model with four electrodes and 99.87% for the case with five electrodes.

## Discussion

In this paper we constructed and analyzed various situations of electric field distribution in the tumor with nearby vessels of different size, orientation and position with respect to tumor tissue and the electrodes. The first aim was to determine in what measure vessels influence the distribution of the electric field, or more precisely, what error occurs in the model of electroporation-based treatments if the vessels are ignored and the consequence of that error on tumor treatment. The second aim was to determine if the automatic segmentation method of hepatic vessels developed in our previous work [22] is robust enough to be used as a part of an automatic treatment planning procedure. In other words, we examined if the known and estimated error of the automatic segmentation method would negatively affect the coverage of the tumor with a sufficiently high electric field.

The results of the first part of the study show that ignoring the presence of a hepatic vessel near a tumor indeed can influence the electric field distribution in such a way that the coverage of the tumor with a sufficiently high electric field decreases. These findings are in accordance with a recent publication by Golberg et al. [35] which studied the effect of nearby blood vessels on cell survival during IRE ablation and found that the presence of vessels near target tissue causes so-called “electric field sinks”. Moreover, we have found that whether the tumor will or will not be sufficiently covered (above 99.9%) directly depends on the tumor size, vessel size and distance between tumor and the vessel. According to the results of this study, for ECT of small tumors (10 mm diameter) the vessels that will have a negative effect are all vessels larger than 3 mm in diameter and less than 3 mm away from the tumor. For ECT of medium-sized tumors (30 mm diameter), all vessels larger than 7 mm in diameter and less than 5 mm away from the tumor will have a negative effect. Vessels are not expected to have any negative effect on the ECT of large tumors (50 mm in diameter).

In the case of IRE, the negative effect of vessels is observed only for IRE of small tumors (10 mm diameter) performed with four electrodes outside the tumor, i.e. without any central electrodes. Observing both IRE and ECT with and without the central electrode unanimously confirms that an electrode configuration with one electrode in the center is more robust and should be the preferred choice in electroporation-based treatments in organs with a high contrast between the conductivity of normal and tumor tissue. It should however be noted that insertion of the central electrode has been raising certain concerns in the medical community, namely regarding the added risk of blood vessel puncture and potential to seed tumor cells along the electrode track upon removal. Regarding the risk of blood vessel puncture, based on the reports of the surgeons that have performed the ECT on the liver one electrode that partially punctures the vessel does not have a critical effect on the vessel structure. Such electrode configuration which would include an electrode that completely punctures the vessel or several electrodes partially puncturing it is something that can be avoided through careful treatment planning. As for the potential of tumor cell seeding upon electrode removal, given the fact that the electric field has the highest values in the close vicinity of the electrodes [34] it would seem theoretically unlikely that any tumor cells would survive in the area near electrodes. However, since we have received reports from several clinicians performing electroporation-based treatments about noticing tumor reseeding along the track of removal of the central electrode (personal communication) it would be wise to perform further investigations of the matter.

The results of the second part of the study confirm what was already shown in the first part: ignoring the vessels while optimizing the treatment can result in tumor under-treatment. Another conclusion the results of studies performed on real patient models confirm is that a large vessel close to tumor without any central electrodes represents a risk of not covering a tumor with a sufficiently high electric field, leading potentially to under-treatment. According to our results, using a central electrode compensates the negative effects that the average anticipated error of the automatic vessel segmentation method (which is 1 pixel) can have on tumor coverage. It is however still possible for the algorithm to produce an error up to 3 pixels, which can negatively affect the tumor coverage even if the central electrode is present. An error of such magnitude is rare and when it does appear it is of smaller scope than we modeled in our experiments, i.e. it is present on usually one side of the object while we introduced it on the whole object edge. Nevertheless, a mechanism to remove this error should be ensured by presenting the results of the automatic vessel segmentation to the clinician or a radiologist for validation before the start of the calculations of the electric field distribution. In case such large error appears the user should be able to spot it relatively easy and can then correct it.

In general, this paper was motivated by the aim to observe the relationship between tumor size, vessel size, and vessel distance between the tumor and the vessel. We aimed at determining the effect of the combinations of these three parameters on the distribution of the electric field and consequently, on the coverage of the tumor with a sufficiently high electric field. We expected that the negative effect of the vessel will be larger for smaller tumors. We also expected that the negative effect of the vessels will be proportional to vessel size and inversely proportional to distance between tumor and vessel. These expectations were confirmed by the results, but not completely. Some anomalies were noted in the cases where the vessels were larger than the tumors and the electrodes were going through the vessels, namely for tumors of 10 mm diameter. The observed anomalies were such that they could negatively affect the success of the electroporation-based treatment, indicating the treatment planning procedure should also be mindful of the mutual position of the electrodes and vessels, not only electrodes and the tumor. This observation increases the level of complexity of an already extremely complex model. With such complexity, the best approach for a successful electroporation-based treatment should necessarily include a treatment planning procedure performed on the whole three-dimensional model.
